# Internet Use, Electronic Health Literacy, and Hypertension Control among the Elderly at an Urban Primary Care Center in Thailand: A Cross-Sectional Study

**DOI:** 10.3390/ijerph18189574

**Published:** 2021-09-11

**Authors:** Auswin Rojanasumapong, Wichuda Jiraporncharoen, Nopakoon Nantsupawat, Mary Ellen Gilder, Chaisiri Angkurawaranon, Kanokporn Pinyopornpanish

**Affiliations:** Department of Family Medicine, Faculty of Medicine, Chiang Mai University, Chiang Mai 50200, Thailand; auswin17@gmail.com (A.R.); wichudaj131@gmail.com (W.J.); nopakoonmd@gmail.com (N.N.); mellietyros@gmail.com (M.E.G.); chaisiri.a@cmu.ac.th (C.A.)

**Keywords:** internet use, electronic health literacy, hypertension, older adults

## Abstract

This study aimed to explore the internet usage and electronic health literacy (eHL) among adults aged 60 and older with hypertension and to explore the associations between eHL and blood pressure control. A cross-sectional survey was conducted at an out-patient primacy care clinic in the urban city center of Chiang Mai, Thailand. eHL was measured using the eHealth Literacy Scale (eHEALS). Logistic regression was used to identify the association between eHL and blood pressure, adjusting for age and sex as a priori confounders and key sociodemographic factors previously identified in univariable analysis. A total of 110 older adult patients with a history of diagnosed hypertension agreed to participate. The mean age of the participants was 67 years old. Fifty-six participants (50.9%) had used the internet in their lifetime. Among internet users, 37 out of 56 participants (66%) could be classified as having high eHL. However, there was insufficient evidence for associations among internet use, eHL and hypertension control. This result potentially creates new opportunities for eHealth education and interventions. Efforts to produce centralized clear, reliable health information targeting this demographic would be worthwhile to help manage chronic diseases such as hypertension in Thailand in the future.

## 1. Introduction

The older adult population in the world is increasing. According to the World Health Organization (WHO), the proportion of people older than 60 increased from 8.5% in 1975 to 12.2% in 2015 [1]. In Thailand, the proportion of people over 60 also increased from 8.7% in 2000 to 15.2% in 2020 [2]. The older adult population faces various medical issues and is especially at risk for chronic disease. Hypertension is a chronic disease that affects up to 52–70% of the older adult population [3,4]. The number of diagnosed patients increased from 600 million in 1980 to nearly one billion in 2008, with 16% of cases being uncontrolled [5,6,7,8]. In Thailand, according to the 2014 Thai National Health Examination Survey [3], 19.7% of hypertension cases are uncontrolled, and 25.47% of hypertension cases in older adult patients are uncontrolled. The global burden of disease due to uncontrolled hypertension is increasing, leading to many consequences including stroke and ischemic heart disease [9,10].

In many developed countries, the internet is a widely used public tool for finding health information [11,12], and electronic health (eHealth) interventions have proven to be beneficial for reducing behavioral risk factors for hypertension such as physical inactivity, and unhealthy diet [13], as well as even controlling hypertension [14,15,16]. Following the widespread limitation of movement and reduction in nonurgent clinic visits due to the COVID-19 pandemic this year, telemedicine has increasingly been used in many countries to protect both patients and healthcare workers [17].

For patients to effectively use the internet for health information and for eHealth interventions, they need some level of eHealth literacy. Electronic health literacy (eHL) is defined as the degree to which individuals have the capacity to obtain, process, and understand basic health information from electronic resources and services needed to make appropriate health decisions [18]. In many developing countries, the proportion of internet access and usage is not as high as in developed countries [19,20], and there are limited data on the level of eHL among the older adult population in developing countries. The lack of internet usage and the uncertain level of eHL may limit the benefits of using the internet for health information and eHealth interventions for chronic disease among older adults.

This study aimed to explore the internet usage and eHL among an older adult population with hypertension in Thailand and to explore the associations between eHL and blood pressure control. 

## 2. Materials and Methods

### 2.1. Design and Study Population

A cross-sectional survey was conducted at the outpatient clinic of the Department of Family Medicine, Faculty of Medicine, Chiang Mai University. The outpatient clinic is a primary care clinic situated in the urban city center of Chiang Mai Province, the largest province in northern Thailand. The clinic is located in the city center of Chiang Mai in northern Thailand where the prevalence of hypertension is about 33% in both urban and rural areas [21]. This clinic is a University Hospital clinical and accepts patients without restrictions. However, most patients coming to the clinic reside near the hospital in Chiang Mai and are mainly government workers and retired personnel. Therefore, most of them are middle-aged, have higher than secondary education, and have regular income, whether through working for the government or having a pension from the government [22].

Between January 2016 and March 2016, our screening nurses invited older adult patients (age 60 and older) with a history of diagnosed hypertension (with ICD-10 diagnosis—with medication or in the process of lifestyle modification) to participate in the survey before seeing the doctor. Walk-in patients were recruited using a convenience sampling method. All participants fitting the inclusion criteria were invited until the target sample size was reached. The sample size calculation was done by using the infinite population proportion formula. According to a previous study, the proportion of older adults with a high level of eHL is 0.463 [23]. Thus, the estimated sample size was about 96. The exclusion criterion was an inability to communicate in the Thai language; however, no one was excluded from our study. Written informed consent was obtained from all participants. The self-administered questionnaire was completed by each patient. Two trained research nurses, who were not part of routine care, were on-site to help clarify any questions from the patients regarding the questionnaire.

### 2.2. Data Collection

#### 2.2.1. Sociodemographic Characteristics

Sex, age, occupation, education, income, and current health status were obtained from a self-administered questionnaire.

#### 2.2.2. Internet Usage and Experience in Using the Internet for Health Information

A questionnaire on internet use was adapted from the Health Information National Trends Survey [24]. Following the WHO process of translation and adaptation of instruments protocol [25], forward and backward translation was done. Content validity was approved by a local expert, and the questionnaire was piloted using 10 older adults. Data on experience in internet use and experience in internet use for health issues were collected. To assess experience in internet use for health issues, we used the following simple question: “Have you used the internet in the last 12 months for health or medical information for yourself?” Participants who answered yes to the question “Do you ever go online to access the internet or worldwide web, or to send and receive e-mails?” were categorized as using the internet.

#### 2.2.3. Electronic Health Literacy (eHL)

eHL was measured using the eHealth Literacy Scale (eHEALS), designed by Norman and Skinner [26] to assess consumers’ perceived skills in using information technology for health and to aid in determining the fit between eHealth programs and consumers. Permission to use and translate the tool into Thai was obtained from the original authors. It was forward-translated, back-translated, and pretested following the WHO process of translation and adaptation of instruments protocol [25]. The final back-translation was sent to the original authors for final approval.

The eHEALS has 10 questions. The first two items are related to the patient’s perception of the usefulness and the importance of the internet for information regarding their health. The next eight items are measures of eHL with respect to the consumer’s combined knowledge, comfort, and perceived skills in finding, evaluating, and applying eHealth information to health problems [25] using a five-point response scale (from 1, “strongly disagree”, to 5, “strongly agree”). For each item, the responses were categorized into three groups: “disagree” (score of 1–2), “undecided” (score of 3), and “agree” (score of 4–5) [27]. Items 3–10 were used to categorize eHL levels. As suggested by the literature, we divided eHL into three levels: (1) never used the internet (for which an eHEALS score was not obtained), (2) low eHL (agreed to <5/8 eHEALS items), and (3) high eHL (agreed to ≥5/8 eHEALS items) [28].

#### 2.2.4. Blood Pressure Control

Blood pressure was measured while sitting upright after resting for 2 min by a nurse with an automatic sphygmomanometer (Model BP-203RVIII, Omron Healthcare Co., Kyoto, Japan). Participants were divided into two groups on the basis of blood pressure: controlled (SBP < 150 and DBP < 90) and uncontrolled (SBP ≥ 150 or DBP ≥ 90) [29].

### 2.3. Statistical Analysis

Sociodemographic data and levels of eHL are presented using descriptive statistics. Factors associated with eHL were examined using the chi-square or Fischer exact test. We used logistic regression to identify the association between eHL and uncontrolled blood pressure, adjusting for age and sex as a priori confounders and key sociodemographic factors previously identified in univariable analysis (*p* < 0.1). All analyses were conducted using STATA 15.1 (Stata Corp LCC, College Station, TX, USA).

## 3. Results

A total of 110 older adult patients with a history of diagnosed hypertension agreed to participate. The mean age of the participants was 67 years old (SD 5.23). A total of 63 were female (57.3%), 55 participants (50%) had a bachelor’s degree or higher, 88 participants (80%) were retired, and 91 participants (82.7%) had a personal income under 30,000 THB per month (845 USD). Among the participants, 56 (50.9%) had used the internet in their lifetime (Table 1).

### 3.1. Electronic Health Literacy

Table 2 shows each eHEALS items with the responses from the 56 participants (50.9%) who had used the internet. Overall, 47 participants (83.9%) reported that the internet was useful in helping them make health decisions, 47 participants (83.9%) felt that it was important for them to be able to access health resources on the internet, and 44 participants (78.6%) felt that they knew how to find useful information on the internet. However, there was a slight drop off in the proportion of participants feeling that they had the skills to evaluate the internet resource (33 participants, 58.9%) and the skills to distinguish between high-quality and low-quality resources (27 participants, 48.2%). Overall, 33 out of 56 older adult internet users (58.9%) felt confident that they could use the information from the internet to make health decisions.

### 3.2. Internet Use, Electronic Health Literacy Level, and Blood Pressure Control

Table 3 shows factors associated with internet use and health literacy. The mean eHEALS score (from items 3–10) was 29.6 (SD 4.15) (ranging from 12–39, with a maximum possible score of 40). Among internet users, 37 participants (66%) could be classified as having high eHL (agreed or strongly agreed with 5–8 items). Men and those with higher income and higher education were more likely to have a higher level of eHL. Not surprisingly, the use of more electronic devices in the past 12 months was associated with higher levels of eHL. There was insufficient evidence to suggest that internet use and eHL were associated with improved blood pressure control in the crude (Table 4) or the adjusted analyses (Table 5).

## 4. Discussion

Around half of the older adult patients attending an urban primary care clinic in Chiang Mai, Thailand had used the internet. Being male, having a higher personal income, having a higher education, and using a higher number of electronic devices in the past 12 months were associated with eHL. However, there was insufficient evidence of an association among internet use, eHL, and hypertension control.

This study showed that half of the participant group had previously used the internet (50.9%). A survey by the National Statistical Office of Thailand estimated that 10.3% of Thais over the age of 60 had previously used the internet [30]. The higher prevalence of internet use in our sample is likely due to the higher education level associated with living in an urban area, home to several universities. However, internet use among the older adults in our sample is still lower than that in other age groups (85.9% of Thais aged 15–24 use the internet; 73.6% for age 25–34 and 61.4% for age 6–14) [31]. Nevertheless, evidence from Thailand also suggests that internet use by the older adults is growing because of the increasing accessibility of internet (and electronic devices). According to National Statistical Office of Thailand, in 2011, only 0.86% of the older adult population used the internet. This number increased to 1.0% in 2012, 3.2% in 2015, and eventually reached 10.3% in 2018. This suggests that the population that can be reached with eHealth interventions appears to be increasing nationally. The potential benefit of eHealth interventions for health management in older adults according to a previous study [32] is the provision of important information in the era of respiratory pandemics which pose a threat to older adults attending primary care visits for chronic illnesses.

The average eHEALS score among those who had used internet in this study was 29.6 (SD 4.15). This is similar to other studies conducted in the United States where the average eHEALS score was reported to be between 29.0 and 30.9 (SD 5.8–6.0) [33,34]. Studies conducted in Japan reported an average eHEALS score between 23.4 and 23.5 (SD 6.4–6.5) [35,36]. While it may not be proper to compare the crude score of eHEALS across studies, it is worth noting that, in addition to variations in demographics and different educational and socioeconomic backgrounds, the internet culture and availability of eHealth resources may also influence eHL among different populations. For example, it has been hypothesized that Japanese have a lower average eHEALS than people living in the United States due to the lack of centralized reliable internet sources of health information in Japanese (e.g., comparable to MedlinePlus from the US National Library of Medicine) [37].

We found that male sex, higher income, higher education, and use of a higher number of electronic devices were associated with higher eHL. Prior studies from the US indicated that women tend to use internet for health purposes more than men [38,39]. However, our study is in line with the 2016 Thailand Internet Use Profile Report. According to the report, men spend a higher number of hours on the internet on average than women [38]. Other associated factors were also in line with previous studies. A prior study from the United States found that having a lower income was associated with lower internet usage, which also led to lower confidence in using internet to find health information [40]. The association between the number of electronic devices used and higher self-perceived eHL was also reported in prior studies [34,40]. Experience with the use of various electronic devices to access health information might increase confidence in using computers, thus leading to improved eHL [41].

In our study, we found no evidence for associations between eHL and blood pressure control; however, previous evidence has suggested that higher eHL is associated with healthy behaviors linked to blood pressure control, such as increased physical activity [36,42,43,44] and eating habits [36,43]. There are many possible explanations for the lack of associations between eHL and blood pressure control in our study. First, only half of our participants had ever used the internet. Evidence suggests that older adult populations in Southeast Asia frequently gain health information from other media, such as television, newspapers, and radio broadcasts, while the internet is one of the least popular methods [45,46]. Second, another study from Thailand suggested that older adult individuals tend to access commercial websites and social media for their health information [43]. These commercial and social media websites are easier to access and draw more attention from audiences but often contain unreliable content, usually promoting the purchase of health products and sometimes distorting correct health information [47]. This might imply that there is a gap between the subjectively perceived eHL and the objective eHL [48]. Older people with a high perceived eHL may not actually obtain, process, and understand online health information to a sufficient level to make decisions on health care. Although the Thai version of the eHEALS tool was found to have good validity [49], a validation study using an objective measure of eHL should be performed. Third, it is possible that reliable health information websites in Thai are not easily accessible or suitable for older adults. Even in developed countries, it has been suggested that suitable resources to develop and promote eHL among older adults are difficult to find [34]. Lastly, a higher eHL may not be associated with better health-promoting behaviors. Many factors may be mediators of blood pressure control such as medication adherence, diet, and regular exercise, which were not explored in our study.

The study was not without its limitations. This was a cross-sectional study, which limits causal interpretation. This study was conducted in one urban outpatient department and used convenient sampling, which may limit generalizability to other settings. Further prospective multisite studies examining the relationship among electronic health literacy, electronic health interventions, and hypertension control are still needed. Although we used a standard questionnaire to assess eHL, i.e., eHEALS, it could only indirectly measure eHL through self-reporting. Thus, it may not directly measure actual skills and knowledge when using the internet for health purposes [50].

## 5. Conclusions

A much higher proportion of older Thai adults in the study population used the internet compared to national surveys, potentially creating new opportunities for eHealth education and interventions. However, we did not find evidence for associations between eHL and blood pressure control. More studies are needed to examine eHL and eHealth interventions in older adults.

## Figures and Tables

**Table 1:** Sociodemographic characteristics (*n* = 110).

Characteristics	*n*	col%
Sex		
Male	47	42.7
Female	63	57.3
Age group		
Old (60–69)	85	77.3
Very old (70 or older)	25	22.7
Body mass index		
<18.5 kg/m^2^	2	1.8
18.5–22.9 kg/m^2^	18	16.4
23–24.9 kg/m^2^	28	25.4
≥25 kg/m^2^	62	56.4
Other underlying disease		
Dyslipidemia	41	37.3
Diabetes mellitus	18	16.4
Antihypertensive medication use		
No medication use	6	5.5
Single pill use	57	51.8
Multiple drugs/combined pill use	47	42.7
Education		
Primary to middle school	34	30.9
Senior high school/high vocational certificate	21	19.1
Bachelor’s degree or higher	55	50.0
Occupational status		
Currently working	22	20.0
Retired	88	80.0
Marital status		
Single/widowed or living alone	32	29.1
Living with spouse/partner	78	70.9
Personal income in THB/month (USD)		
<10,000 (<280)	26	23.6
10,000–30,000 (280–845)	65	59.1
>30,000 (>845)	19	17.3
Ever used the internet		
No	54	49.1
Yes	56	50.9

Abbreviation: THB, Thai baht; USD, US dollars.

**Table 2:** Electronic Health Literacy Scale (eHEALS) scores among internet users (*n* = 56).

*Questions*		*row%*	
	Disagree (Score1–2)	Undecided (Score3)	Agree (Score4–5)
How useful do you feel the internet is in helping you make decisions about your health?	0.0	16.1	83.9
2. How important is it for you to be able to access health resources on the internet?	1.8	14.3	83.9
3. I know what health resources are available on the internet.	5.4	21.4	73.2
4. I know where to find useful health resources on the internet.	1.8	26.8	71.4
5. I know how to find useful health resources on the internet.	7.1	14.3	78.6
6. I know how to use the internet to answer my questions about health.	1.8	37.5	60.7
7. I know how to use the internet information I find to help me.	3.5	17.9	78.6
8. I have the skills I need to evaluate the health resources I find on the internet.	7.1	33.9	58.9
9. I can tell high-quality health resources from low-quality health resources.	5.4	46.4	48.2
10. I feel confident in using information from the internet to make health decisions.	1.8	39.3	58.9

**Table 3:** Factors associated with electronic health literacy (*n* = 110).

Characteristics	No Internet Use (*n* = 54)	Low eHL (*n* = 19)	High eHL (*n* = 37)	*p*-Value
Sex				
Male	44.4	15.8	54.1	0.022
Female	55.6	84.2	45.9	
Age group				
Old (60–69)	70.4	79.0	86.5	0.194
Very old (70 or older)	29.6	21.0	13.5	
Body mass index				
<18.5 kg/m^2^	3.7	0	0	0.198
18.5–22.9 kg/m^2^	24.1	15.8	5.4	
23–24.9 kg/m^2^	22.2	21.0	32.4	
≥25 kg/m^2^	50.0	63.2	62.2	
Other underlying disease				
Dyslipidemia	38.2	31.6	32.2	0.629
Diabetes mellitus	11.1	21.1	21.6	0.343
Antihypertensive medication use				
No medication use	5.6	15.8	0	0.103
Single pill use	57.4	36.8	51.3	
Multiple drugs/combined pill use	54.0	47.4	48.7	
Education				
Primary to middle school	42.6	15.8	21.6	0.071
Senior high school/high vocational certificate	20.4	21.1	16.2	
Bachelor’s degree or higher	37.0	63.2	62.2	
Occupational status				
Currently working	72.2	89.5	86.5	0.130
Retired	27.8	10.5	13.5	
Marital status				
Single/widowed or living alone	68.5	63.2	78.4	0.427
Living with spouse/partner	31.5	36.8	21.6	
Personal income in THB/month (USD)				
<10,000 (<280)	33.3	15.8	13.5	0.029
10,000–30,000 (280–845)	59.3	52.6	62.2	
>30,000 (>845)	7.4	31.6	24.3	
Number of electronic devices used (col%)				
None	9.3	5.3	0.0	<0.001
1	88.9	53.1	54.1	
2 or more	1.8	31.6	45.9	

**Table 4:** Internet use, electronic health literacy, medication use, and blood pressure control (*n* = 110).

		Controlled Blood Pressure (BP < 150/90)		Uncontrolled Blood Pressure (BP ≥ 150/90)		*p*-Value
**Ever Used the Internet**		***n***	**col%**	***n***	**col%**	
	No	37	48.1	17	51.5	0.739
	Yes	40	51.9	16	48.5	
**Electronic Health Literacy**		***n***	**col%**	***n***	**col%**	
	No internet use	37	48.0	17	51.5	0.642
	Low eHL	15	19.5	4	12.1	
	High eHL	25	32.5	12	36.4	

Abbreviation: eHL, electronic health literacy.

**Table 5:** Association between electronic health literacy and uncontrolled blood pressure (*n* = 110).

Uncontrolled Blood Pressure (BP ≥ 150/90)	Adjusted OR ^a^	95% CI	*p-*Value
No internet use experience	Reference		
Low eHL	0.47	0.12 to 1.92	0.292
High eHL	0.90	0.31 to 2.59	0.840

Abbreviation: CI, confidence interval; eHL, electronic health literacy; OR, odds ratio. ^a^ Adjusted for age, sex, education income, and medication use.

## Data Availability

Data are available from Chiang Mai University Ethics Committee (contact via researchmed@cmu.ac.th) for researchers who meet the criteria for accessing confidential data.
